# Gut mucosal cells transfer **α**-synuclein to the vagus nerve

**DOI:** 10.1172/jci.insight.172192

**Published:** 2023-12-08

**Authors:** Rashmi Chandra, Arpine Sokratian, Katherine R. Chavez, Stephanie King, Sandip M. Swain, Joshua C. Snyder, Andrew B. West, Rodger A. Liddle

**Affiliations:** 1 Department of Medicine,; 2 Department of Pharmacology and Cancer Biology,; 3 Department of Surgery, and; 4 Department of Cell Biology, Duke University, Durham, North Carolina, USA.; 5 Duke Institute for Brain Sciences, Durham, North Carolina, USA.; 6 Aligning Science Across Parkinson’s (ASAP) Collaborative Research Network, Chevy Chase, Maryland, USA.; 7 Department of Veterans Affairs, Chevy Chase, Maryland, USA.

**Keywords:** Gastroenterology, Neuroscience, Parkinson disease

## Abstract

Epidemiological and histopathological findings have raised the possibility that misfolded α-synuclein protein might spread from the gut to the brain and increase the risk of Parkinson’s disease. Although past experimental studies in mouse models have relied on gut injections of exogenous recombinant α-synuclein fibrils to study gut-to-brain α-synuclein transfer, the possible origins of misfolded α-synuclein within the gut have remained elusive. We recently demonstrated that sensory cells of intestinal mucosa express α-synuclein. Here, we employed mouse intestinal organoids expressing human α-synuclein to observe the transfer of α-synuclein protein from epithelial cells in organoids to cocultured nodose neurons devoid of α-synuclein. In mice expressing human α-synuclein, but no mouse α-synuclein, α-synuclein fibril-templating activity emerged in α-synuclein–seeded fibril aggregation assays in intestine, vagus nerve, and dorsal motor nucleus. In newly engineered transgenic mice that restrict pathological human α-synuclein expression to intestinal epithelial cells, α-synuclein fibril-templating activity transfered to the vagus nerve and dorsal motor nucleus. Subdiaphragmatic vagotomy prior to induction of α-synuclein expression in intestinal epithelial cells effectively protected the hindbrain from emergence of α-synuclein fibril-templating activity. Overall, these findings highlight a potential non-neuronal source of fibrillar α-synuclein protein that might arise in gut mucosal cells.

## Graphical abstract

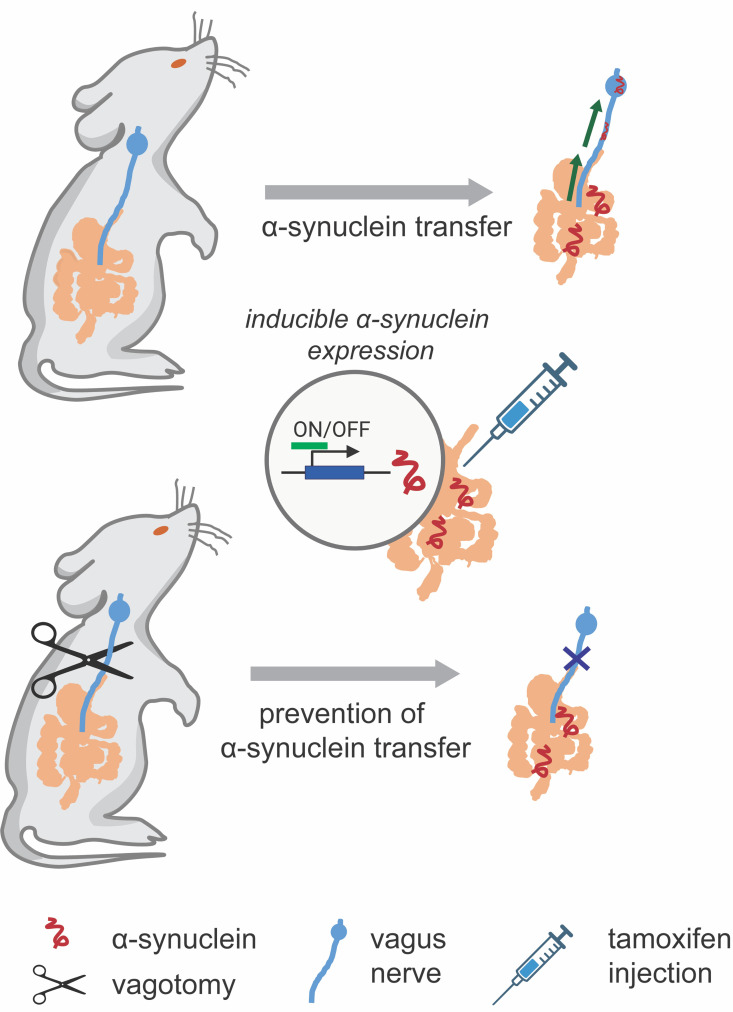

## Introduction

Parkinson’s disease (PD) is a debilitating neurodegenerative disease with characteristic motor disturbances including rigidity, resting tremor, and bradykinesia. Many patients also suffer from gastrointestinal symptoms such as constipation that often precede characteristic motor deficits by 10 years or more (1). The pathological hallmarks of PD are intracellular proteinaceous inclusions filled with fibrillated forms of α-synuclein that accumulate in both the brain and peripheral nervous system. In dopaminergic neurons in PD, inclusions known as Lewy bodies have been associated with neuronal vulnerability and degeneration (2, 3). Throughout the brain, α-synuclein is normally found in presynaptic terminals of excitatory neurons and other neuronal subtypes, with a role in endocytosis and synaptic vesicle function (4). Mutations in the α-synuclein gene (*SNCA*) such as A53T and A30P, as well as multiplication of the *SNCA* locus, can cause familial PD (5, 6). One of the remarkable features of α-synuclein protein is the intrinsic ability to aggregate into β-sheet–rich protein fibrils that have high affinity toward amyloid dyes like thioflavin (7–9). These α-synuclein fibrils have a proposed capacity to spread between interconnected cells in a hypothesized prion-like cascade (10–13). Transferred α-synuclein might recruit native α-synuclein within the recipient cell to seed additional aggregates (14–16) that can form larger fibrils and inclusions (17, 18). The presence of α-synuclein fibrils and templating activity in the brain and cerebrospinal fluid has been convincingly demonstrated in PD as measured through newly developed seeded aggregation assays that include protein misfolding cyclic amplification and real-time quaking-induced conversion (RT-QuIC) assays (19). The α-synuclein RT-QuIC assay demonstrated seeding activity in duodenal biopsies of patients with PD but not in healthy control individuals (20). The originating source of the α-synuclein seeds that trigger activity in this assay are not clear. In rat models, it is thought that seeds that trigger pathological accumulations of α-synuclein may originate in neurons and the brain and descend into the gut or originate somewhere in the gut and ascend into the brain (21).

Clinical and experimental data indicate that the gut may play a role in PD susceptibility. Not only do gastrointestinal symptoms such as constipation often precede the motor symptoms of PD (22–24), experimentally, it has been suggested in rodent models that exogenous α-synuclein fibrils introduced into the gut can spread to the brain (25–27). Abnormal α-synuclein aggregates have been histopathologically identified in the enteric nervous system prior to the development of PD (22). Evidence supporting a role for the enteric nervous system involvement in α-synuclein pathology includes the observation that α-synuclein immunoreactive inclusions localize to neurons of the submucosal plexus, whose axons project to the gut mucosa (28–30) and myenteric plexus, and parallel input of the vagus nerve (31). Mice harboring the A53T transgene exhibited enteric nervous system dysfunction including increased colonic transit times and reduced fecal output consistent with constipation (32, 33). In animals, the vagal route of α-synuclein transport has also been documented following exposure to the environmental toxicant rotenone that can cause α-synuclein misfolding (34), as well as direct injections of adeno-associated viral vectors overexpressing human α-synuclein in vagal neurons (35). More recently, it was demonstrated that vagotomy prevented the formation of brain aggregates when α-synuclein fibrils were injected into the gut of susceptible mice, indicating that pathogenic fibrils can spread from nerve terminals in the gut to the brain (25, 36). However, the physiologically relevant origins of misfolded α-synuclein protein that might use the vagus nerve as a conduit into (or out of) the brain have been unclear. One clue might be that some epidemiological observations suggest that complete truncal vagotomy in patients is associated with a decreased risk of PD, suggesting in at least some PD cases that α-synuclein aggregation in the gut may be critical for PD risk later on (37–39).

Recently, it was discovered that enteroendocrine cells (EECs) in the gut mucosa connect with neurons in culture and in the intact gut of mice (40). Using modified rabies viral tracing in vivo, a synaptic connection was verified between peptide YY–expressing EECs and neurons in the colon (40). In coculture experiments in dishes, cholecystokinin-containing (CCK-containing) EECs and sensory neurons form spontaneous synaptic connections (40, 41). Thus, there appears to be an inherent affinity between EECs and neurons (42). EECs are exposed to the lumen of the gut and respond to numerous chemical and physical stimuli from diet, the microbiome, and the environment (40, 43–45). Long thought to produce exclusively gut hormones, it is now known that EECs possess neuron-like properties, and part of that neuron-like phenotype might include the endogenous expression of α-synuclein (42, 46). EECs express neurotransmitters and other canonical presynaptic proteins and possess axon-like processes sometimes called neuropods through which they connect to nearby nerves (40, 41). To explore a potential cellular source of pathological α-synuclein that might contribute seeding activity from the gut to the brain, here we use organoids and transgenic mice to explore whether α-synuclein has a potential to spread from gut mucosal cells to the vagus nerve.

## Results

**Figure 1:**
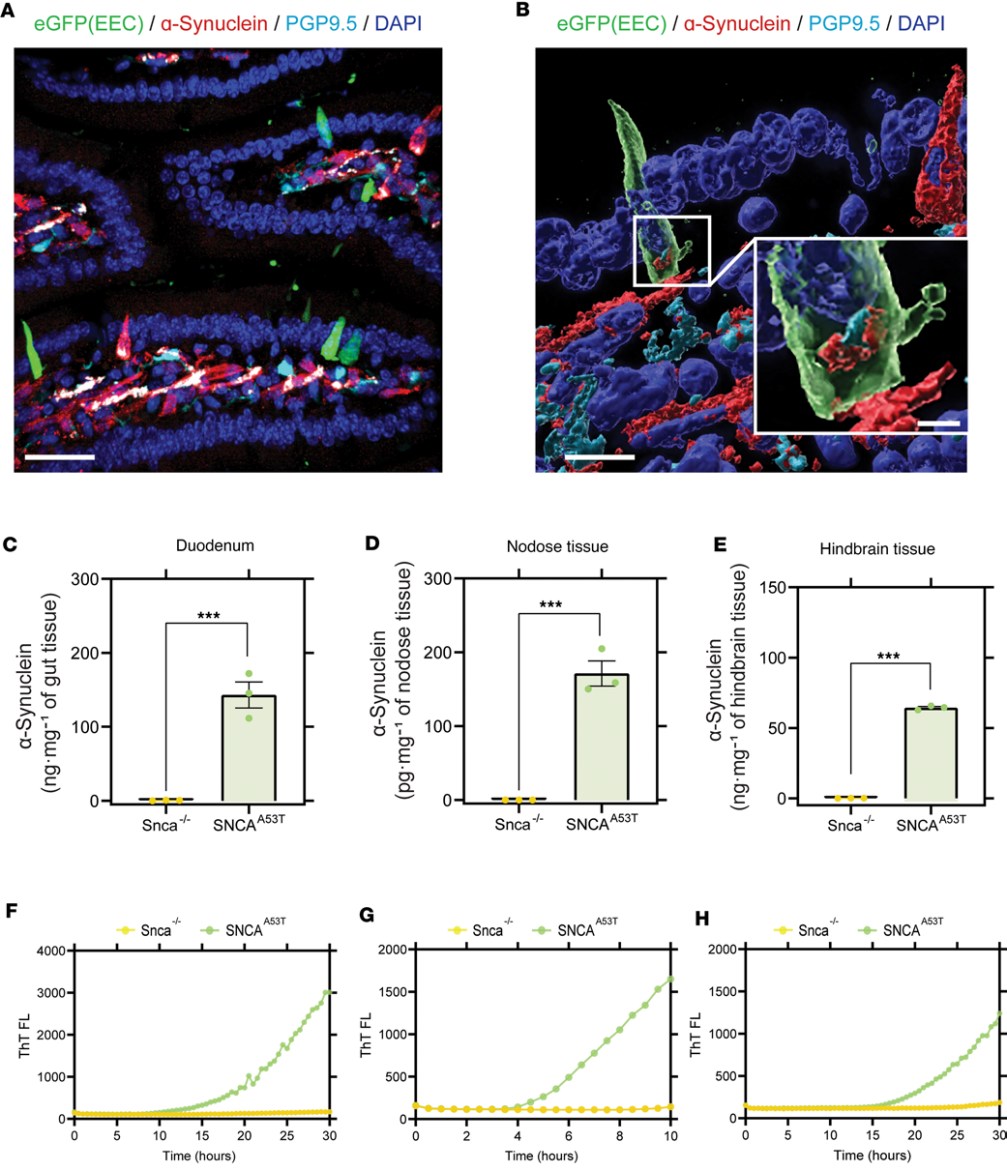
α-Synuclein expression and seeding activity in SNCA^A53T^ mice. (**A** and **B**) Immunostaining of duodenum harvested from SNCA^A53T^ mice. Enteroendocrine cells (EECs) expressing green fluorescent protein (shown in green) are scattered among other mucosal cells (DAPI-labeled nuclei, blue) and are in proximity to α-synuclein–containing (red) fibers stained with the pan-neuronal marker PGP9.5 (cyan) in the lamina propria of the villus. ELISA quantification of human α-synuclein in (**C**) duodenum (α-synuclein quantification in ng/mg of nodose tissue), (**D**) nodose ganglia (α-synuclein quantification in pg/mg of nodose tissue), and (**E**) hindbrain (α-synuclein quantification in ng/mg of nodose tissue), from *Snca*^–/–^ and SNCA^A53T^ mice. RT-QuIC analysis of (**F**) duodenum, (**G**) nodose ganglia, and (**H**) hindbrain of *Snca*^–/–^ and SNCA^A53T^ mice. Scale bars are 30 μm (**A**), 10 μm and 1 μm (inset) (**B**). All the group analyses are shown as mean ± SEM. All RT-QuIC curves shown are representative of the mean from all the groups analyzed. Significance was determined by unpaired *t* test; ***P < 0.001. *n* = 3.

In the gut, EECs present as elongated or flask-shaped cells and express high levels of α-synuclein protein according to our past immunohistochemical analyses (46). The apical surface of EECs is typically open to the intestinal lumen, and the basal surface lies on the lamina near neurons (Figure 1A). To study the possible spread of pathological (i.e., human A53T α-synuclein) α-synuclein protein, we established a humanized α-synuclein mouse model, in which endogenous *Snca* was deleted and replaced with α-synuclein protein expressed from a P1-derived artificial chromosome (PAC) encoding human A53T α-synuclein (SNCA^A53T^). This mouse strain is known to develop mild but widespread pS129-α-synuclein protein accumulations with physiological levels of α-synuclein levels (32, 47) and develops α-synuclein immunoreactive aggregates in enteric ganglia by 3 months of age as well as early gastrointestinal dysfunction (32). Cck-eGFP mice (which express EGFP in EECs), also devoid of endogenous mouse α-synuclein, were crossed with mice expressing the PAC-Tg(*SNCA^A53T^*) (48) to generate PAC-Tg(*SNCA^A53T^* Cck-*eGFP* *Snca^–/–^*). This mouse line is referred to hereafter as SNCA^A53T^. SNCA^A53T^ mice develop normally and exhibit human α-synuclein expression that localizes primarily to the basal pole of EECs in the gut (Figure 1B). Cck-eGFP expression demarcates some EECs in the proximal intestine (40, 41). Vagal innervation of the gut is most dense in the proximal intestine and diminishes in the colon (49); therefore, to investigate the potential neuronal spread of α-synuclein, we focused on the small intestine. According to sensitive ELISA analysis for human α-synuclein protein, α-synuclein expression in the vagal nodose ganglia was low (~170 pg/mg of tissue, Figure 1C) compared with much higher expression in the duodenum and upper intestine (~140 ng/mg of tissue, Figure 1D), with lesser expression in the hindbrain that encompasses the dorsal motor nucleus (Figure 1E). Despite the much lower levels of α-synuclein protein in nodose ganglia compared with the hindbrain, the isolated ganglia tissue harbored positivity for fibril-seeding activity in RT-QuIC assays (dwell time to threshold 5.79 hours/1.43 hours, Figure 1F). Dwell times in RT-QuIC reactions indicated possible femtomolar concentrations of α-synuclein fibril seeds in the lysates from the nodose tissue according to standard curves of recombinant fibrils of known concentrations (Supplemental Figure 1; supplemental material available online with this article; https://doi.org/10.1172/jci.insight.172192DS1). Comparable RT-QuIC signals in gut and hindbrain tissues were also observed corresponding to dwell times to threshold of 19.1 hours/5.5 hours (Figure 1, G and H). Matched tissues dissected and processed at the same time from the same region in the mice that lacked human α-synuclein expression did not reveal any comparable fibril-templating activity (Figure 1, F–H). These results extend the observations of Kuo et al., who originally demonstrated the presence of enteric α-synuclein pathology with immunohistochemistry in the gut in the PAC-Tg(*SNCA^A53T^*) mouse strain (32).

**Figure 2:**
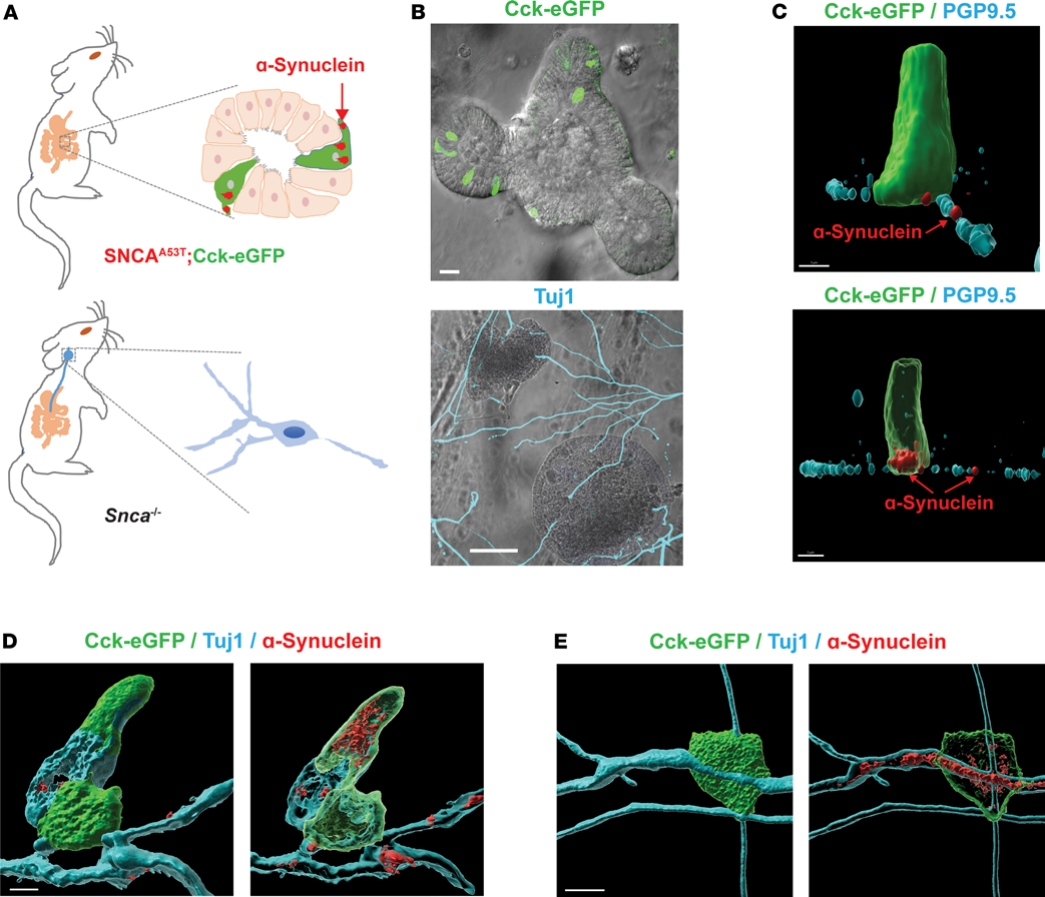
Human A53T α-synuclein protein transfers from gut cells to adjoining vagal neurons. (**A**) Intestinal organoids were prepared from an SNCA^A53T^ mouse in which CCK-containing cells express enhanced green fluorescent protein (eGFP), and vagal nodose ganglia neurons were isolated from an *Snca*^–/–^ mouse lacking endogenous α-synuclein. (**B**) Representative images of organoids and neurons grown in coculture for 5 days, with eGFP-positive cells (green) in the organoid and β-tubulin III (Tuj1, cyan) highlighting neuronal processes. (**C**) Representative high-magnification α-synuclein (red) staining of an eGFP-positive EEC. Red arrow indicates localization to a PGP9.5-positive (cyan) process in an *Snca*^–/–^ mouse neuron. (**D** and **E**) Representative images with neuron-specific β-tubulin III (cyan). Surface and (adjacent) intracellular confocal slices are shown. Scale bars are 30 μm for **B**, 3 μm for **C**, and 5 μm for **D** and **E**.

Recognizing that pathological α-synuclein is expressed in EECs in the model, and EECs lie in close proximity to enteric submucosal neuronal fibers, we next sought to determine if α-synuclein expressed in EECs might spread to adjacent nerves. To evaluate this possibility, we cocultured intestinal organoids from the SNCA^A53T^ mice with nodose ganglia neurons from *Snca^–/–^* mice that lack any α-synuclein expression (Figure 2). Though enteric ganglia might natively express low levels of α-synuclein protein (Figure 1C), culturing the neurons from the *Snca^–/–^* mice and utilizing a monoclonal anti-human α-synuclein antibody previously validated in the *Snca^–/–^* mice (50) ensure reliability for detection of authentic α-synuclein spread from the intestinal organoids to the cocultured nerves. In the organoids, the EECs (identified by eGFP expression) are oriented with their apical surface open to the lumen (Figure 2B). Given that organoids remain stationary within the Matrigel matrix, it appeared that nerve fibers with strong PGP9.5 or neuron-specific Tuj1 expression from the *Snca^–/–^* mice grew toward the basal surface of EECs, demonstrating that organoids attract and connect with nerve fibers in vitro. Such results are consistent with previous observations with isolated EECs (40, 41, 51). As expected, neuronal fibers from *Snca^–/–^* mice exhibited no detectable α-synuclein via immunohistochemistry (Supplemental Figure 2). Within 5 days, some nerve fibers had grown toward organoids and appeared to be in close contact with EECs (i.e., CCK-positive cells, Figure 2, C–E). CCK-positive EECs from SNCA^A53T^ mice (shown in green) in intestinal organoids express readily detectable α-synuclein (shown in red). More striking was the stark appearance of α-synuclein protein within PGP9.5 (a pan-neuron-specific marker) neuritic outgrowths nearby CCK-eGFP–positive cells (Figure 2C). While α-synuclein protein distributed in the cytoplasm of EECs, α-synuclein protein colocalized within neurites from *Snca^–/–^* mice in patches along the process, presented as patches along the neuritic PGP9.5-positive length. Transferred α-synuclein protein could be detected along PGP9.5 processes on the basal organoid surface running next to EEC cells (Figure 2C). α-Synuclein protein was similarly observed in Tuj1-positive neurites in which neuronal fiber staining appeared more uniform (Figure 2, D and E). Because of the high densities inherent to the organoid systems, and high connectivity and dynamic outgrowth of cocultured ganglia, it was not possible to ascertain a critical distance that facilitated the transfer of α-synuclein onto adjacent neuronal fibers, even though other studies in 2D chambered cells suggest direct cell-to-cell contact is required for transfer (42). α-Synuclein protein appeared to emerge from basal and lateral regions of the EECs that were also in physical contact with PGP9.5- or Tuj1-positive ganglionic outgrowths.

**Figure 3:**
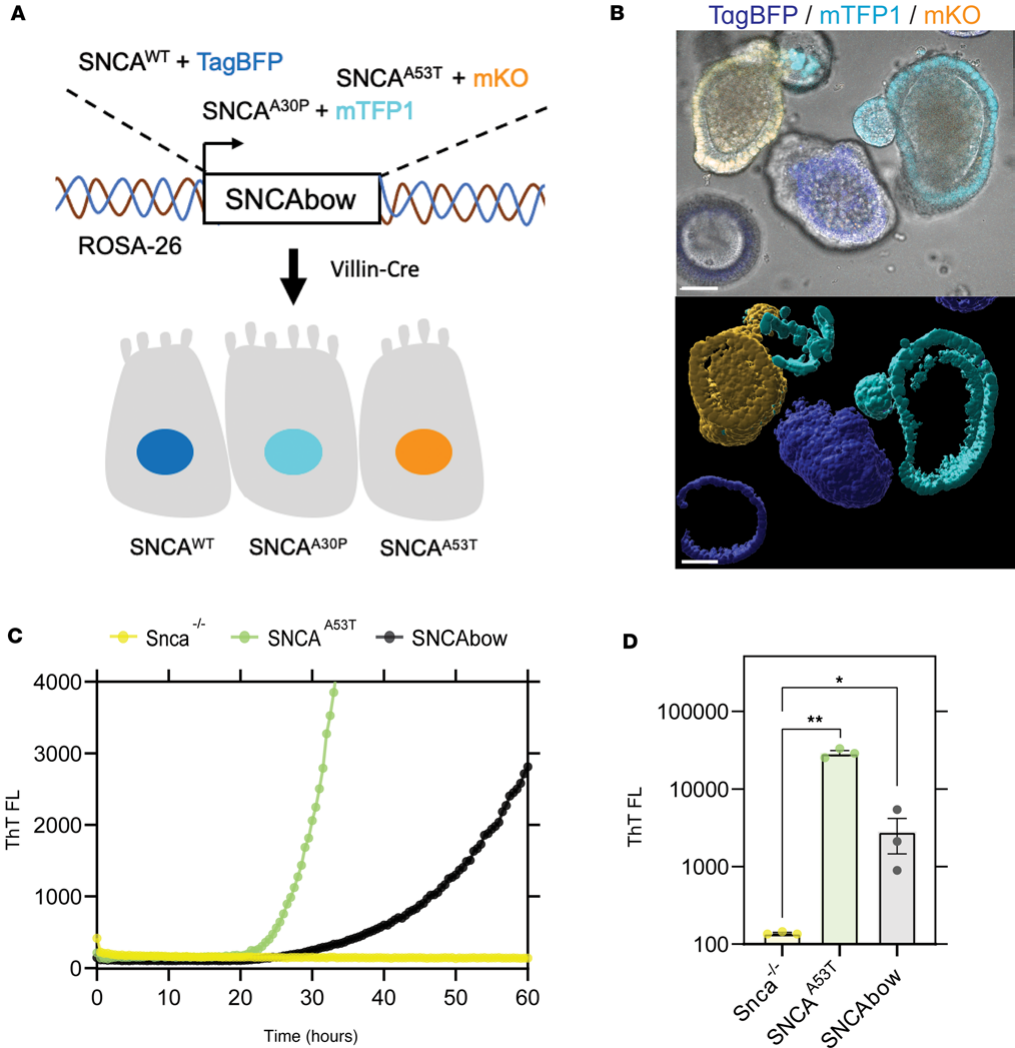
Conditional human α-synuclein expression induces α-synuclein seeding activity in gut organoids. (**A**) The SNCAbow expression construct contains 4 tandem cassettes downstream of a chicken β-actin promoter (not shown). The first cassette (not shown) expresses a chemically inducible near-infrared fluorogen-activating peptide (FAP-Mars1). The next 3 cassettes encode a unique fluorescent protein (TagBFP: blue, mTFP1: cyan, or mKO: orange) and a corresponding human synuclein protein SNCA^WT^, SNCA^A30P^, and SNCA^A53T^. When Tg mice are mated to the *Villin*-Cre (Vil-Cre) strain, Cre-mediated recombination by 3 pairs of orthogonal lox sites (LoxN, Lox2272, LoxP) results in the expression of a single fluorescent protein marker and the corresponding human α-synuclein in any given mucosal cell. (**B**) Photomicrograph of a small intestine organoid illustrates 3 fluorescent proteins in the mucosa of an SNCAbow mouse indicating the expression of SNCA^WT^ TagBFP (blue), SNCA^A30P^ mTFP1 (turquoise), and SNCA^A53T^ mKO (orange). Scale bar = 30 μm. (**C** and **D**) RT-QuIC endpoint ThT fluorescence analysis of nodose ganglia from *Snca^–/–^*, SNCA^A53T^, and SNCAbow mice at 6 months of age. (**C**) A representative ThT fluorescence profile for these genotypes is provided. (**D**) Endpoint values were collected after 100 hours of RT-QuIC relative to negative controls. Data were collected and combined from 3 mice for each strain in triplicate. All the group analyses are shown as mean ± SEM. All RT-QuIC curves shown are representative of the mean from all the groups analyzed. Significance was determined by 1-way ANOVA with a Dunnett’s post hoc analysis relative to *Snca^–/–^*; *P < 0.05, **P < 0.01, *n* = 3.

To determine if gut-to-neuron α-synuclein transfer might occur in intact mammals, and the possible distance to which transferred α-synuclein protein might travel along ganglionic processes (e.g., to the dorsal motor nucleus), we developed a Tg strategy to overexpress human wild-type and mutant α-synuclein isoforms exclusively in the gut mucosa. This approach is based on *Villin*-Cre conditional expression. Not knowing if aggregation-prone mutated human α-synuclein (i.e., A53T) or wild-type protein may be more likely to transfer from the EECs to nerves, we utilized a recently described “Crainbow” mouse modeling approach to express pathological *SNCA* gene variants in the same tissue (52). We fluorescently barcoded 3 forms of human *SNCA* (wild-type, A30P, and A53T mutants) with coexpressed spectrally resolvable fluorescent proteins from a ROSA-targeting vector for generating α-synuclein-Crainbow mice that we refer to as SNCAbow mice (Figure 3A and Supplemental Figure 3). SNCAbow mice encode human wild-type α-synuclein coexpressed with nuclear TagBFP (SNCA^WT^ TagBFP), SNCA^A30P^ coexpressed with nuclear mTFP1 (SCNA^A30P^ mTFP1), and SNCA^A53T^ coexpressed with nuclear mKO (SNCA^A53T^ mKO). In the absence of Cre recombinase activity, only a near-infrared FAP is expressed. Crossing SNCAbow mice with Vil-Cre mice results in recombination and expression of SNCA^WT^ TagBFP, SNCA^A30P^ mTFP1, or SNCA^A53T^ mKO (Figure 3A). From this gene construct, a fluorescent protein and the corresponding α-synuclein were produced as a single mRNA transcript with 2 distinct polypeptides upon translation (blue TagBFP/SNCA^WT^, turquoise mTFP1/SNCA^A30P^, and orange mKO/SNCA^A53T^). We validated expression using intestinal organoids that demonstrate expression of all 3 fluorescent colors (blue, turquoise, and orange), indicating all 3 human α-synuclein genes (wild-type, A30P, A53T) were expressed specifically in the intestinal mucosa (Figure 3B and Supplemental Figure 4). Interestingly, some cultured organoids expressed only 1 color, while some organoids expressed multiple colors. This pattern of expression is consistent with clonal growth typical in many organoids where 1 cell type can dominate others in expansion (53). We have previously observed that in the gut mucosa cells, α-synuclein appears exclusively in EECs as observed by immunohistochemistry (46). Without addition of external stimulus, preformed α-synuclein fibrils, and so on, α-synuclein fibril-templating activity was detected in the gut organoids in culture nearly comparable to that observed in organoids cultured from the SNCA^A53T^ mice (Figure 3, C and D). In contrast, organoids matched from *Snca^–/–^* mice lacked any observable seeding activity.

**Figure 4:**
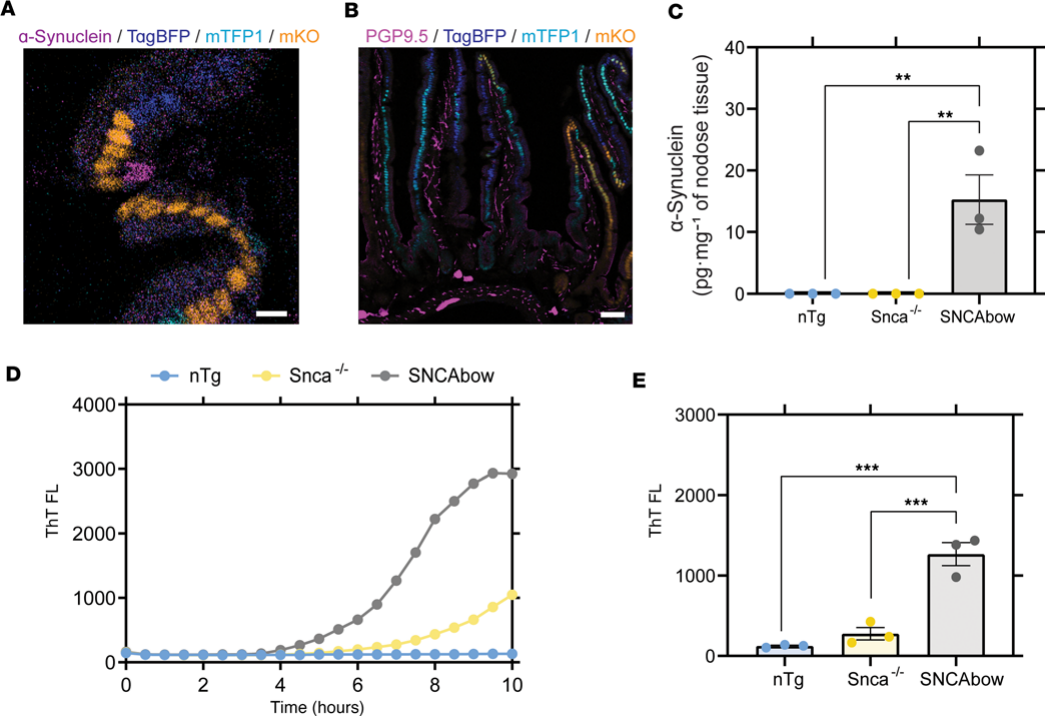
Conditional human α-synuclein expression in gut mucosal cells produces in α-synuclein seeding activity in nodose ganglia. (**A**) SNCAbow mouse duodenum illustrating expression of fluorescent protein markers BFP (blue), TFP (turquoise), and mKO2 (orange) in mucosal cells (enterocytes and EECs). α-Synuclein (magenta) immunostaining is present in a single intestinal mucosal cell consistent with an EEC (scale bar = 10 μm). (**B**) Duodenum from SNCAbow mouse illustrating PGP9.5-positive neuronal fibers (magenta) innervating intestinal crypts and villi (scale bar = 50 μm). (**C**) ELISA quantification of human α-synuclein protein in nodose ganglia of nontransgenic, *Snca^–/–^*, and SNCAbow mice. (**D**) A representative ThT fluorescence profile (RT-QuIC) and endpoint analysis of nodose ganglia from nontransgenic (nTg), *Snca^–/–^*, and SNCAbow mice at 1 month of age. (**E**) RT-QuIC analysis of nodose ganglia from 6-month-old nTg, *Snca^–/–^*, and SNCAbow mice. All the group analyses are shown as mean ± SEM. All RT-QuIC curves shown are representative of the mean from all the groups analyzed. Significance was determined by 1-way ANOVA with a Dunnett’s post hoc analysis relative to SNCAbow; **P < 0.01, ***P < 0.001, *n* = 3.

In the intestine of SNCAbow mice, with α-synuclein protein expression directed by *Villin*-Cre expression, α-synuclein protein was detected in gut mucosal cells via immunofluorescence (Figure 4, A and B) but not in submucosal enteric nerves. We have previously demonstrated that prominent α-synuclein immunostaining in EECs is easily visualized in SNCA^A53T^ mice, which contain 4 copies of the human α-synuclein transgene and express at physiological levels in the gut (46). However, a sensitive ELISA approach for human α-synuclein protein (that does not cross-react with mouse α-synuclein protein) detected the presence of human α-synuclein protein transferred to the vagus of SNCAbow mice (Figure 4C). Notably, the level of α-synuclein presumably transferred from *Villin*-Cre–expressing epithelia (including EECs) in the gut (15.27 ± 4 pg/total mg of protein) was about one-tenth the level of α-synuclein detected in ganglia in the PAC-human α-synuclein Tg mouse (Figure 1C). Despite the lower levels, α-synuclein present in the vagus nerve presumably transferred from gut *Villin*-Cre–expressing cells; the RT-QuIC method for measuring fibril-templating activity resolved robust fibril-templating activity in the SNCAbow vagus (Figure 4, D and E). In contrast, no significant templating activity could be identified in nontransgenic mice with only normal concentrations of mouse α-synuclein in the vagus. These results suggest that pathological, templating-positive α-synuclein protein seeds might originate in gut cells in the SNCAbow Tg mice that transfer α-synuclein protein to the vagus nerve that otherwise lacks intrinsic pathological α-synuclein expression. It was not possible to discern the proportion that each expressed α-synuclein gene (wild-type, A30P, A53T) contributed to the RT-QuIC signal in the vagus.

**Figure 5:**
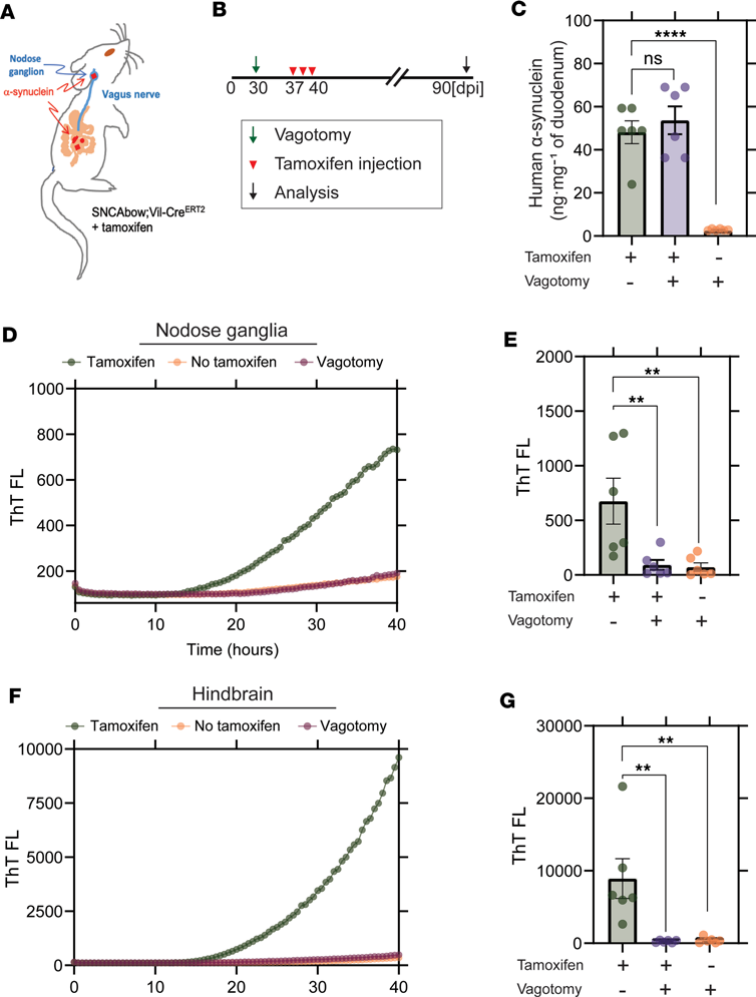
Vagotomy spares the nodose ganglia from α-synuclein seeding activity and prevents spread to the hindbrain. (**A** and **B**) Experimental model. SNCAbow Vil-Cre^ERT2^ mice underwent bilateral subdiaphragmatic vagotomy or sham surgery 1 week before tamoxifen treatment. (**C**) ELISA measurements of α-synuclein protein in the gut 3 months after tamoxifen treatment. RT-QuIC analysis of (**D** and **E**) vagal nodose ganglia and (**F** and **G**) hindbrain analyzed 3 months after tamoxifen treatment. Representative ThT fluorescence profiles are shown in **D** and **F**. All the group analyses are shown as mean ± SEM. All RT-QuIC curves shown are representative of the mean from all the groups analyzed. Data points in **B**, **E**, and **G** represent the significance determined by a 1-way ANOVA with a Dunnett’s post hoc analysis relative to tamoxifen-treated and no-vagotomy groups. **P < 0.01, ****P < 0.0001, *n* = 6.

We next tested whether a prophylactic subdiaphragmatic vagotomy procedure might protect the vagus, and ultimately the hindbrain, from the proposed pathological transfer of α-synuclein protein from gut cells. For timed inducible expression of α-synuclein in the gut of the vagotomized adult mice, we crossed the SNCAbow mouse with a tamoxifen-inducible Cre recombinase (SNCAbow Vil-Cre^ERT2^) to allow aging and subdiaphragmatic vagotomy procedures prior to induction of any human α-synuclein expression (Figure 5, A and B). Three months after tamoxifen treatment, α-synuclein expression was evident in the intestine that was unchanged with vagotomy (Figure 5C). Fibril-templating activity in dissected vagal ganglia was again detected by RT-QuIC after tamoxifen treatment (Figure 5, D and E). In contrast, templating activity was not detected for the mice that underwent the vagotomy procedure or mice that did not receive tamoxifen treatment. These results suggest that pathological α-synuclein fibril activity likely spreads from the gut cells of the SNCAbow mice to the hindbrain via the vagus nerve (Figure 5, F and G).

## Discussion

This study explores the potential for gut mucosal cells to contribute pathological α-synuclein protein to the nervous system from gut cells. Included are data from 3 experimental systems: mixed organoid-neuron cocultures, SNCAbow mice, and inducible SNCAbow mice with or without vagotomy. Collectively, the data indicate that pathological human α-synuclein can transfer from gut mucosal cells to interconnected nerves in mouse models. The main limitations of the study include the reliance on transgenic α-synuclein expression in the gut, as well as a lack of separation of the effects of mutated human pathological α-synucleins from wild-type α-synuclein protein. Future studies may include the creation of new mouse strains that separate different α-synuclein isoforms and expression levels in this process. Further, it remains unclear the molecular nature of the cell-to-cell transfer that might be occurring, whether through bulk exocytosis and uptake (54–57) or a more refined local cell-to-cell process like tunneling nanotubes that exist in neuron-to-neuron and glial connections but have yet to be resolved in gut-to-brain signaling (58–61). The organoid-neuron coculture system provided initial evidence that pathological α-synuclein could transfer from EECs but was limited as an in vitro model. However, verifying that α-synuclein spread also occurs in vivo in SNCAbow mice supports the relevance of the coculture system, which could be used to explore the cellular mechanisms of EEC-to-neuron transfer. Nevertheless, these results highlight the existence of a non-neuronal potential source for pathological α-synuclein protein that may contribute to the pool of abnormal α-synuclein at some point in disease.

Braak et al. highlighted the hypothesis that PD pathology may arise in peripheral nerves and spread to the central nervous system in some cases (22). The identification of α-synuclein aggregates in enteric nerves, before their appearance in the brain, may be consistent with gut-to-brain pathological spread (29, 62). However, according to prion-like activity associated with α-synuclein in disease (especially in models of disease), and α-synuclein cell-to-cell transfer observed in different paradigms, corrupted and misfolded α-synuclein need not originate in the cells that wind up with large observable aggregates. Based on the evidence presented herein, we hypothesize that the gut mucosa, especially EECs, may contribute pathological misfolded α-synuclein to vulnerable efferent and afferent projections of the vagus nerve, which might predispose to the risk of Lewy body disease. Notably, EECs in the mucosa make physical contact with both the microbiome and ingested toxicants like pesticides in the gut lumen, as well as contacting nerve fibers on the other side, providing a ripe opportunity to better understand the pathobiology of the microbiome and toxin exposures in PD vulnerability.

Vagotomy is known to abolish the spread of gut-injected recombinant preformed fibrils (PFFs) to the brainstem, indicating that the transmission mechanism might occur via the vagus nerve (25). Herein, pathological α-synuclein was introduced not through the injection of preformed α-synuclein fibrils but through the overexpression of either wild-type or mutated human α-synuclein isoforms in gut mucosal cells. According to ELISA analysis, the overexpression achieved was modest and in line with PAC-Tg expression that has been described as physiologically comparable to α-synuclein expression in humans (32). Although EECs are distributed throughout the gastrointestinal tract, we focused on the proximal small intestine, where vagal innervation is most abundant.

Due to their high sensitivity, seeding amplification assays have been used with increasing frequency to detect early pathology in PD and distinguish patients with PD from nonaffected individuals (63, 64). We used RT-QuIC to detect the early spread of α-synuclein to obtain a semiquantitative estimate of α-synuclein abundance in tissue lysates. This approach is similar to human seeding assay results for tissues like duodenum and cerebrospinal fluid (20, 65).

Transplants of gut microbiota from patients with PD into gnotobiotic mice accelerate α-synuclein pathology and PD-associated behavioral changes in mice bearing human α-synuclein (66). The mechanism of formation of pathological α-synuclein in response to the imbalanced gut microbiota remains elusive, albeit we hypothesize here the role of EECs as a possible target given that they are directly exposed and respond to microbes and microbial metabolites (67, 68). Recently it has been demonstrated that *Akkermansia muciniphila*, a bacterial strain residing in the gastrointestinal tract and associated with PD, led to α-synuclein aggregation in an enteroendocrine cell line (69). Therefore, although the specific habitat for pathological α-synuclein in the gut is unknown, our identification of α-synuclein in EECs and the location of EECs at the interface between the gut lumen, rich with microbiota and enteric nerve fibers, has raised the possibility that EECs may be a source for the formation and possible spread of pathological α-synuclein (46). Alternatively, pathological α-synuclein from the brain may spread into EECs that might then transfer to interconnected neurons otherwise devoid of pathological α-synuclein. The initial step in determining whether EECs are capable of transmitting pathological α-synuclein was verified by our in vitro studies demonstrating the transfer of human A53T α-synuclein from EECs in organoid culture onto isolated *Snca^–/–^* nodose ganglion neurons. Using nerves lacking endogenous α-synuclein together with an antibody specific for human α-synuclein, it was possible to establish that EECs were the source of the α-synuclein immunoreactivity appearing in the nearby neurons. This experiment demonstrated that EECs possess the ability to transfer endogenous α-synuclein onto adjacent nerves, a process that could be the initial step in the spread of pathological α-synuclein into the nervous system in some patients in disease. Despite the direct transfer of α-synuclein from EECs to nodose neurons in vitro, whether other cell types contribute to the spread in vivo is unknown. EECs come into contact with glia (45) and connect to enteric neurons (40) as well as the vagus nerve (41). It is possible that α-synuclein can spread to enteric glia (70) and, thus, provide a potential route for the spread of α-synuclein. It remains to be determined what role glia might have in the spread of α-synuclein to enteric nerves including the vagus that extends beyond the gut.

EECs are sensory cells of the intestine that communicate directly with afferent vagal nerve fibers (41). The function of α-synuclein expression in these cells remains to be clarified. The close proximity of EECs to the nerve processes prompted us to look for α-synuclein in the vagus in the gut-restricted models. Our detection of α-synuclein seeding activity in the vagus in the SNCAbow mice is consistent with reports of neuropathology in the sensory branch of the vagus nerve in patients with PD (71) and the possible spread of PFFs to the nodose ganglia in experimental models of PD (27). However, it is not known if this is a common route for the spread of PD.

It is generally believed that templating of α-synuclein in the recipient cell is necessary to facilitate cell-to-cell propagation of pathological α-synuclein. It is notable in this regard that α-synuclein protein does not appear to reside in the dendrites of vagal afferents, where it would be available for such templating (72). Nevertheless, the in vitro coculture experiments demonstrated that α-synuclein in the recipient neuron is not required for α-synuclein uptake. The appearance of α-synuclein templating activity in SNCAbow nodose ganglia in vivo suggests that the Tg human α-synuclein can spread at least as far as the vagal ganglia. Therefore, it is plausible that α-synuclein may transfer to vagal afferents and spread intracellularly before any templating with endogenously expressed protein occurs in the axonal compartment of the neuron. The question of whether transmission occurring along vagal afferents, or descending back to the gut from the brain, preferentially drives disease phenotypes in disease is a major question that can be tackled in part in the future with additional conditionally restricted α-synuclein expression models.

## Methods

### Mice.

Mice expressing EGFP in CCK cells [Tg(Cck-EGFP)BJ203Gsat/Mmmh], referred to as Cck-eGFP, were obtained from Mutant Mouse Resource and Research Center (RRID:MMRRC_000249-MU), and the colonies were maintained on a Swiss Webster background (Taconic Biosciences) (73). FVB;129S6-*Snca^1nbma^* Tg(SNCA*A53T)1Nbm *Snca*^tm1Nbm^Tg(SNCA*A53T)2Nbm/J mice (PAC-*SNCA*^A53T^) (32) and *Snca*^–/–^ mice (74) were obtained from Robert L. Nussbaum, University of California, San Francisco, San Francisco, California, USA (RRID:IMSR_JAX:010799). The generation of *SNCA*^A53T^ Cck-eGFP mice has been described previously (46) (referred to herein as SNCA^A53T^).

Human wild-type, A53T, or A30P forms of α-synuclein were expressed from a modified brainbow gene construct that also harbored 3 fluorescent proteins (blue, turquoise, and orange). In this manner only 1 fluorescent protein could be expressed from each copy of the construct. Cre recombinase is driven by the *Villin* promoter–targeted expression to mucosal cells of the gastrointestinal tract with different fluorescent proteins expressing in each stem cell. SNCAbow mice were generated using a technique that was recently described (52). Briefly, C-terminal tagged α-synuclein genes (SNCA tagged with V5, hSNCA-A30P mutant tagged with 3XHA, and SNCA-A53T mutant tagged with Myc) were amplified by PCR and pENTR plasmids were generated. These plasmids were sequenced in entirety at Massachusetts General Hospital Center for Computational & Integrative Biology DNA Core (https://dnacore.mgh.harvard.edu/new-cgi-bin/listing.action) and cloned by Infusion cloning into a ROSA26 mouse targeting vector adapted for Gateway cloning. Plasmid DNA harvested from bacterial colonies was mapped by restriction enzyme digestion analysis and positive colonies were identified. The entire plasmid was sequenced, linearized with X*ho*I, and transfected into G4 embryonic stem (ES) cells (129/B6N hybrid ES line) (MMRRC catalog MMRRC:011986-UCD, RRID:CVCL_E222). Putative positive ES cell clones were processed and validated across the homology arms by PCR. DNA from 2 selected bacterial colonies was amplified using LA Taq DNA polymerase (TaKaRa), and the region between the homology arms was sequenced. ES cells from the selected clone were microinjected into ICR/Hsd morulae to produce chimeric mice. The Tg mouse was mated with ROSA FLPe [Jax-129S4/SvJaeSor-Gt(ROSA)26Sortm1(FLP1)Dym/J (Jackson Laboratory, RRID:IMSR_JAX:003946)] to excise the neomycin cassette, then mated with *Vil*-Cre mouse [B6.Cg-Tg(*Vil*1-cre)997Gum/J (Jackson Laboratory, RRID:IMSR_JAX:004586) or Tg(*Vil*1-cre/ERT2)23Syr (gift of Sylvie Robine, Institut Curie-CNRS, Paris, France, RRID:IMSR_JAX:020282)] for expression of fluorescent proteins and associated α-synuclein transgenes in intestinal mucosal cells.

### Vagotomy and tamoxifen treatment.

Surgical subdiaphragmatic vagotomy was performed in 1-month-old male and female SNCAbow Vil-Cre^ERT2^ mice. Mice were anesthetized with ketamine (50–100 mg/kg), and an abdominal laparotomy was performed. Immediately below the diaphragm, the vagus nerve was identified and isolated from surrounding connective tissue and vessels. A 2 mm section of the vagus nerve was excised, and the surgical wound was closed with surgical clips. Mice were administered analgesics and observed daily for 5 days for any signs of distress. In sham-operated animals, abdominal laparotomy was performed, and the vagus nerve was exposed but not excised. Weight loss of ~15% was noted in mice undergoing vagotomy compared with sham surgery. One week following the surgery mice were treated with tamoxifen (5 mg/kg) or vehicle administered by intraperitoneal injection daily for 5 days.

### Preparation and culture of mouse organoids.

Mouse small intestine was dissected, gently flushed with ice-cold phosphate-buffered saline (PBS) (pH 7.4)/Primocin (1:1,000) (InvivoGen, catalog ant-pm-1), cut into ~0.5 cm pieces that were placed in 7.5 mL cold PBS/EDTA (3 mM)/Primocin/Y27632 (1:1,000) (ApexBio, catalog A3008-200) containing penicillin-streptomycin (Gibco, catalog 15140-122), and gently shaken for 15 minutes at 4°C. The intestinal tissue was transferred to fresh EDTA/PBS/Primocin/Y27632, shaken for 25 minutes at 4°C, and transferred to PBS. Tissue was then transferred to PBS/Y27632, shaken for 2 minutes, and filtered through a 70 μm mesh, examined under a microscope, and aliquoted at a density of 50 crypts in 15 μL growth factor–reduced Matrigel (Corning, catalog 354230). The suspension was centrifuged at 475*g* for 5 minutes at 4°C, and the pellet was resuspended in cold growth factor–reduced Matrigel and aliquoted (15 μL/well) in a 48-well plate (Eppendorf, catalog 0030723113). Matrigel was left to polymerize for 30 minutes at 37°C. To each well, we added 200 μL of prewarmed (37°C) Intesticult Media (StemCell Technologies, catalog 06005) containing Primocin. Media were changed every 2 days and organoids were split weekly.

### Isolation and coculture of nodose ganglion neurons.

Nodose ganglia were dissected from *Snca^–/–^* mice and placed in 300 μL ice-cold mouse Intesticult Media containing nerve growth factor-2 (NGF) (25 ng/mL, MilliporeSigma, catalog N6009) and liberase (0.156 mg/mL). After incubating at 37°C for 30 minutes, the supernatant was replaced with 500 μL Intesticult Media containing NGF. Tissue was dissociated by pipetting, filtered through a 70 μm mesh, and centrifuged at 211*g* for 2 minutes at room temperature. The pellet was resuspended in fresh media, mixed with growth factor–reduced Matrigel (Corning, catalog 354230), and added to intestinal organoid cultures (at least 4 weeks old). The nodose ganglia/organoid mixture was incubated in an 8-well Chamber slide (Thermo Fisher Scientific) at 37°C for 30 minutes to allow polymerization. Subsequently, prewarmed Intesticult Media containing NGF was added, and the cell mixture were grown for an additional 5–8 days prior to imaging.

### Immunostaining of organoids.

Whole-mount organoid staining was performed as described previously (75) with slight modifications. After removal of media, organoids were fixed in 4% paraformaldehyde in PBS (prewarmed at 37°C to prevent Matrigel depolymerization) for 20 minutes at room temperature. Organoids were permeabilized with prewarmed 0.5% Triton X-100 in PBS, followed by 3 washes with 100 mM glycine (Invitrogen, catalog 10977-023). After blocking with 5% bovine serum albumin/5% donkey serum/PBS for 2 hours at room temperature, primary antibody was added, and incubation continued in a humidified chamber for 16 hours at 4°C. Primary antibodies used for immunostaining included rabbit CCK (73), rabbit α-synuclein (Abcam catalog ab138501, RRID:AB_2537217, at 1:1,000), guinea pig PGP9.5 (Abcam catalog ab10410, RRID:AB_297150, at 1:100), chick β-tubulin III (Tuj1; Neuromics catalog CH23005, RRID:AB_2210684, at 1:100), and chick GFP (Abcam catalog ab13970, RRID:AB_300798, at 1:1,000). Slides were washed 3 times for 20 minutes each in IF buffer (PBS containing 0.1% BSA, 0.2% Triton X-100, 0.05% Tween 20). Secondary antibodies (in IF) were applied for 1 hour at room temperature in the dark. Secondary antibodies included donkey anti-chicken Alexa Fluor 488 (Jackson ImmunoResearch Labs catalog 703-545-155, RRID:AB_234037, at 1:500), donkey anti-mouse Alexa Fluor 568 (Jackson ImmunoResearch Labs catalog 715-006-150, RRID:AB_234037, at 1:500), and donkey anti–guinea pig Alexa Fluor 647 (Jackson ImmunoResearch Labs catalog 706-605-148, RRID:AB_2340476, at 1:250). Slides were washed 3 times, 20 minutes each wash, with IF buffer. DAPI was applied for 5 minutes at room temperature, washed in PBS, and mounted with ProLong Gold (Thermo Fisher Scientific, catalog P36930).

### IF of duodenal and nodose tissue sections.

To characterize expression of transgenes and fluorescent proteins, mice were anesthetized with a mixture of xylazine and ketamine and perfused with ice-cold 3.5% freshly depolymerized paraformaldehyde. Intestinal tissue was harvested, postfixed, and cryopreserved in graded sucrose solutions. The tissue was embedded in OCT, and cryosections (10–20 μm thickness) were collected on Fisherbrand Superfrost Plus Microscope Slides (Thermo Fisher Scientific). Immunostaining was performed as described previously (46). The following primary antibodies were used: chick GFP (Abcam catalog ab13970, RRID:AB_300798, at 1:1,000), rabbit α-synuclein (Abcam catalog ab138501, RRID:AB_2537217, at 1:1,000), sheep α-synuclein (Abcam catalog ab6162, RRID:AB_2192805, at 1:1,000), and rabbit PGP9.5 (MilliporeSigma catalog AB1761-I, RRID:AB_2868444, at 1:50).

### α-Synuclein protein expression and purification.

Plasmid construct (pRK172-human-α-synuclein) encoding wild-type human α-synuclein was expressed in BL21-CodonPlus (DE3-RIL) cells (Agilent, catalog 230245-41). Protein expression was induced with 0.1 mM isopropyl β-d-1-thiogalactopyranoside at cell density OD (600 nm) 0.8 and overnight incubation at 18°C with continuous shaking. Cell pellets were lysed in 0.75 M NaCl, 10 mM Tris HCl pH 7.6, 1 mM EDTA, and 1 mM PMSF and sonicated at 30% power (Thermo Fisher Scientific 500 Dismembrator) for 1 minute followed by boiling the cell suspension 15 minutes. Centrifuged and filtered samples were dialyzed against 10 mM Tris HCl, pH 7.6, with 50 mM NaCl, 1 mM EDTA, and 1 mM PMSF. The suspension was passed through a HiPrep Q HP 16/10 column, 1 × 20 mL, on an ÄKTA pure protein purification system (both Cytiva, formerly GE Healthcare) with running buffer composed of 10 mM Tris pH 7.6 and 25 mM NaCl, then eluted with a linear gradient application of high-salt buffer (10 mM Tris HCl, pH 7.6, 1 M NaCl). Fractions containing a single band of α-synuclein were identified by Coomassie staining of SDS-PAGE gels and were dialyzed and concentrated. Purified monomer protein was subjected to 2 to 3 rounds of endotoxin removal (Endotoxin removal kit, GenScript) until a level of < 0.1 EU/mg was achieved. Endotoxin levels were determined using an LAL chromogenic endotoxin quantification kit (GenScript).

### Preparation of mouse tissue homogenates for RT-QuIC assay.

Mouse tissues (10% weight/volume) were prepared by homogenizing the tissue in PBS supplemented with 1% Triton X-100 followed by probe tip sonication for 1 minute (5 seconds on, 15 seconds off) at 10% amplitude (Thermo Fisher Scientific 500 Dismembrator). Sonicated samples were centrifuged for 20 minutes at 20,000*g* at 4°C, and supernatants were aliquoted for further applications. For RT-QuIC analysis, 1% of homogenates were added into the reaction.

### RT-QuIC.

RT-QuIC analysis was performed in clear-bottom, ultra-low-binding, 384-well plates (Corning) loaded with 1 zirconia silica bead (2.3 mm, OPS Diagnostics) per well. Reaction conditions included 10 μM ThT in sterile PBS, 20 μM of human α-synuclein monomer, and 10% of tissue homogenates (1% weight/volume). Human α-synuclein monomer was passed through an ultra-0.5 centrifugal filtration system (100 kDa MWCO, Amicon) to remove high–molecular-weight aggregates. Reactions were performed in triplicate, and each technical and biological experiment was repeated 3 times. Standard curves with serial dilutions of recombinant α-synuclein fibrils in 1% of matrix homogenate from *Snca^–/–^* mice were included for each reaction in order to confirm the efficiency, sensitivity, and specificity of the reaction (Supplemental Figure 1). Serial dilutions of recombinant fibrils were prepared according to approximate molecular weight calculated via Dynamic Light Scattering measurements and estimated structural arrangements as described (47). Negative control samples including the lysates from *Snca^–/–^* tissues were used to exclude any background signals likely occurring due to tissue origins. The RT-QuIC assay was conducted using an OMEGA BMG plate reader for 15–90 hours with 60 seconds of shaking at 700 rpm and 60 seconds of rest. The ThT signal was monitored every 30 minutes at 448-10 nm excitation and 482-10 nm emission.

### Chemiluminescence-enhanced ELISA measurements for human α-synuclein.

Determination of human α-synuclein concentration in nodose ganglia was performed according to manufacturer’s instructions (BioLegend, catalog 844101). The 1:10 diluted samples were run in triplicates, and reference standard curve ranged 6.1–1,500 pg/mL. Luminescent signal was recorded on a ClarioStar plate reader (BMG).

### Statistics.

Statistical analyses were performed via the GraphPad Prism 9 software. Unpaired 2-tailed *t* test was applied for comparison between 2 groups, and 1-way ANOVA with a Dunnett’s post hoc analysis was performed for multiple groups as indicated in figure legends. Statistical significance was considered with *P* values less than 0.05.

### Study approval.

All experiments were performed with approval by the Duke University Institutional Animal Care and Use Committee.

### Data availability.

Values for all data points found in graphs are in the Supporting Data Values file, which has also be deposited at https://doi.org/10.7924/r4542x03c

## Author contributions

RC and AS contributed to the experimental design, conducted experiments, and wrote the manuscript. KRC, SK, and SMS conducted experiments and reviewed the manuscript. JCS contributed to the experimental design and reviewed the manuscript. ABW contributed to the experimental design and wrote the manuscript. RAL designed the experiments and wrote the manuscript.

## Supplementary Material

Supplemental data

Supporting data values

## Version 1 12/08/2023

Electronic publication
