# Supplementary material for: Gut mucosal cells transfer α-synuclein to the vagus nerve
Source: JCI Insight. 2023 Dec 8;8(23):e172192. doi: 10.1172/jci.insight.172192 (PMC10795834; doi:10.1172/jci.insight.172192)
Supplement: Supplemental data [file jciinsight-8-172192-s085.pdf]

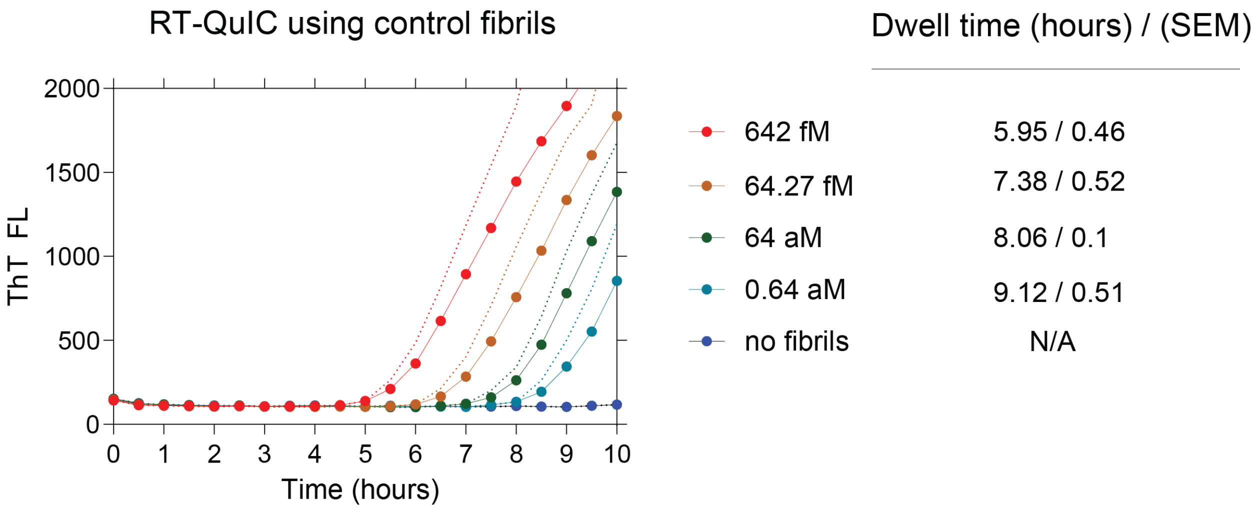

Supplemental Figure 1. Spike-in recombinant fibril seeds demonstrates sensitivity of RT-QuIC assays in protein lysates from *Snca*$^{-/-}$ nodose ganglia lysates

Histograms showing a titration of concentrations of fibril seeds over four orders of magnitude in RT-QuIC reactions. Reaction buffer includes 1% of tissue lysates from nodose ganglia from *Snca*$^{-/-}$ mice to account for matrix effects on α-synuclein aggregation. Dwell times are with respect to increases in ThT 2xSEM. above background (e.g., no fibril) parallel runs. Each dot shows the mean value from triplicate independent reactions (SEM indicated with dashed lines).

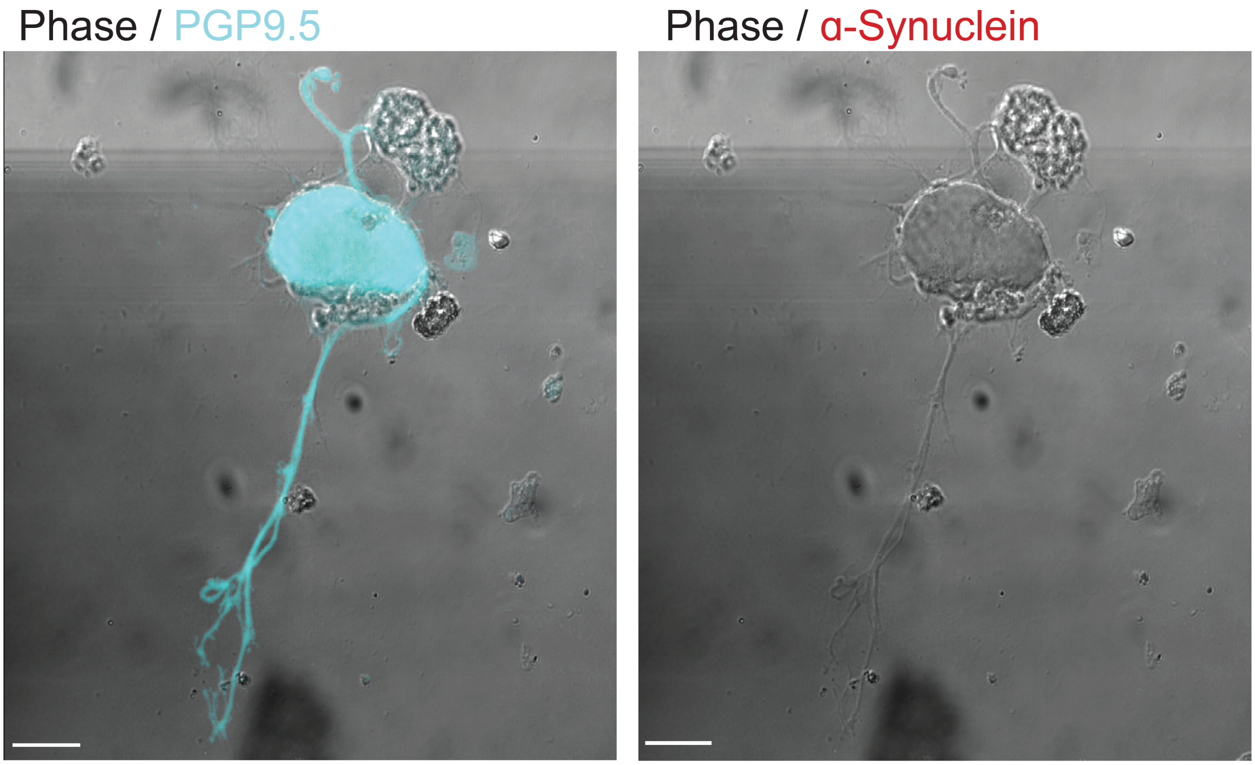

Supplemental Figure 2. Nodose ganglion neuron from *Snca*$^{-/-}$ mouse lacks α-synuclein. (Left panel) Confocal photomicrograph of nodose ganglion neuron that was isolated from *Snca*$^{-/-}$ mouse and grown in culture for one week prior to staining with PGP9.5 antibody (turquoise) (Abcam Cat# ab10410). (Right panel) Immunofluorescence staining with antiserum for α-synuclein (red) (Abcam, ab138501, MJFR1). As expected, neurons from *Snca*$^{-/-}$ mice have no immunodetectable α-synuclein. Bar = 30 μm.

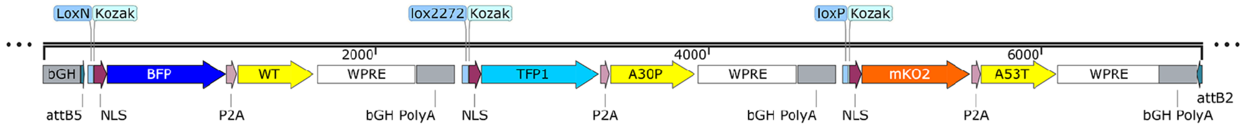

Supplemental Figure 3. SNCAbow construct expresses three human α-synuclein proteins. (A) The SNCAbow expression construct contains four tandem cassettes downstream of a chicken-β-actin promoter (not shown). The first cassette (not shown) expresses a chemically inducible near-infrared fluorogen-activating peptide (FAP-Mars1). The next three cassettes encode a unique fluorescent protein (TagBFP:blue, mTFP1:turquiose, or mKO:orange) and a corresponding human α-synuclein protein $SCNA^{WT}$, $SCNA^{A30P}$, or $SCNA^{A53T}$. When transgenic mice are crossed with mice expressing Cre-recombinase, Cre-mediated recombination by three pairs of orthogonal lox sites (LoxN, Lox2272, LoxP) results in the expression of a single fluorescent protein marker and the corresponding human α-synuclein in any given mucosal cell.

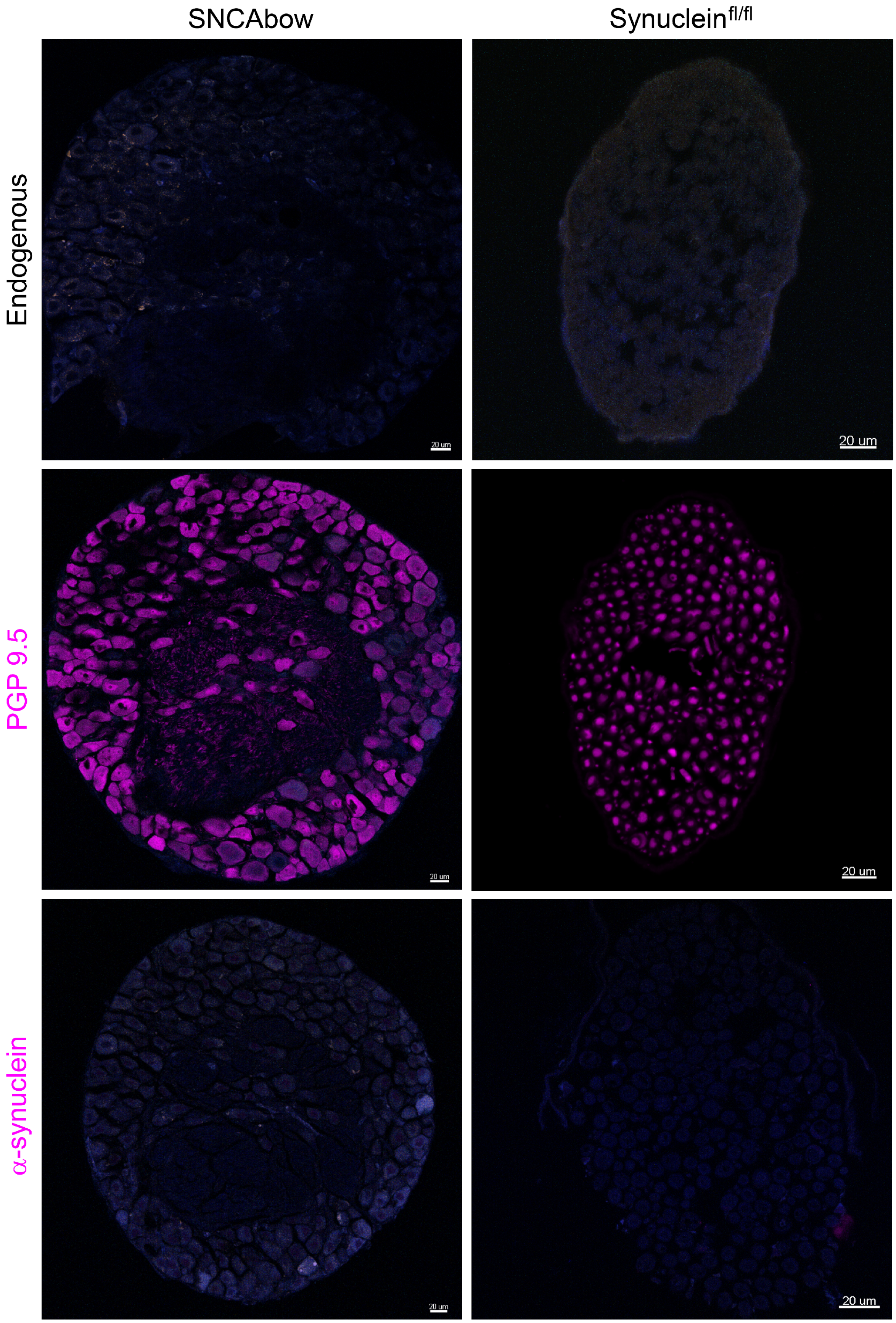

SNCAbow
Synuclein$^{fl/fl}$
Endogenous
PGP 9.5
α-synuclein
20 um
20 um
20 um
20 um
20 um
20 um

Supplemental Figure 4. Nodose ganglia of SNCAbow mice do not express aberrant transgene fluorescence. Confocal images showing endogenous fluorescence (no immunostaining) (top row), PGP9.5 (Millipore Cat# AB1761-I) immunostaining (middle row), and α-synuclein immunostaining (bottom row) in the nodose ganglion of SNCAbow and α-synuclein$^{fl/fl}$ mice (in the absence of Vil-Cre). Unlike the duodenum, endogenous fluorescence was not detected in the nodose ganglion of SNCAbow mice. PGP9.5 immunostaining is present in nerve bundles of both genotypes of mice. α-Synuclein (Abcam, Cat# ab138501, MJFR1) could not be detected.
